# Translational genomics and multi-omics integrated approaches as a useful strategy for crop breeding

**DOI:** 10.1007/s13258-018-0751-8

**Published:** 2018-10-23

**Authors:** Hong-Kyu Choi

**Affiliations:** Department of Molecular Genetics, College of Natural Resources and Life Science, Dong-A University, Nakdong-Daero 550-Beongil 37, Saha-Gu, Busan, 49315 Republic of Korea

**Keywords:** Translational genomics, Omics, Crop breeding, Database, Platform

## Abstract

Recent next generation sequencing-driven mass production of genomic data and multi-omics-integrated approaches have significantly contributed to broadening and deepening our knowledge on the molecular system of living organisms. Accordingly, translational genomics (TG) approach can play a pivotal role in creating an informational bridge between model systems and relatively less studied plants. This review focuses mainly on addressing recent advancement in omics-related technologies, a diverse array of bioinformatic resources and potential applications of TG for the crop breeding. To accomplish above objectives, information on omics data production, various DBs and high throughput technologies was collected, integrated, and used to analyze current status and future perspectives towards omics-assisted crop breeding. Various omics data and resources have been organized and integrated into the databases and/or bioinformatic infrastructures, and thereby serve as the ome’s information center for cross-genome translation of biological data. Although the size of accumulated omics data and availability of reference genomes are different among plant families, translational approaches have been actively progressing to access particular biological characteristics. When multi-layered omics data are integrated in a synthetic manner, it will allow providing a stereoscopic view of dynamic molecular behavior and interacting networks of genes occurring in plants. Consequently, TG approach will lead us to broader and deeper insights into target traits for the plant breeding. Furthermore, such systems approach will renovate conventional breeding programs and accelerate precision crop breeding in the future.

## Introduction

What is ‘translational genomics (TG)’? And how can genetic and/or genomic information be translated across diverse species? TG is possible on the basis of two assumptions. First, the genetic blue prints of all organisms living on the earth are composed of the same chemical language, namely duplexed polynucleotide chains consisting of four different types of nucleotides or simply DNA. Second, all living organisms had originated and diverged from the common ancestor, and have evolved into new species on the basis of DNA change (i.e., mutations), and its expansion, modification and accumulation for an enormous span of time. Based on such idea, we can study many aspects of genome-related disciplines; (1) genome-to-genome comparison, (2) identification of orthologous genes from many different species, (3) phylogenetic analysis and molecular evolution, (4) discovery of trait-associated genes and its practical application to other species.

Conventional crop breeding techniques are based exclusively on phenotypic selection and still mostly practiced in the field of breeding program (Varshney et al. 2015), even if all those processes are still time-consuming and labor-intensive. The ultimate goal of crop breeding aims to achieve a genetic gain of desirable traits into crop genomes in time- and cost-efficient manners. To overcome the drawback of conventional breeding programs, TG approach has recently begun to be introduced in some major crops, such as rice, maize and legumes (Varshney et al. 2015; Lawrence and Walbot 2007).

Before the genomics era opened, translation or transfer of genetic information gained from one species to another was quite restricted mainly due to lack of suitable genomic knowledge. However, the advent of next generation sequencing (NGS) technology during the first decade of the twenty-first century has revolutionized and unprecedentedly accelerated production of genomic and other omics data, thereby leading towards a new era of the biological ‘big data’. Such rapid accumulation of various types of omics data facilitates TG approaches, and translational accuracy will be further improved by the development of more sophisticated bioinformatic tools in the future.

This review mainly focuses on translational genomics and other omics-derived approaches in plants and crops, including current status of recently advanced technologies for massive production of omics data, representative public resources of databases, and strategy and perspectives of TG applications for the crop breeding in the future.

### Bio-big data and translational genomics

One of the most important factors by which can empower TG approaches is technical invention and innovations in sequencing technologies. Dideoxynucleotide-based Chain termination method for DNA sequencing, first developed by Sanger et al. (1977), was based on a fine chemistry, and almost all genome scientists have been dependent on this technology approximately for 40 years, because it was a sole means for acquiring DNA information in the past. But now, such situation has been dramatically changed due to recent advent of NGS technologies in 2007 (Hutchison 2007).

This technical innovation has now exceeded the Moore’s law, resulted in a dramatic reduction of the sequencing costs and accelerated production of sequence data at so called sky-rocketing speed (http://www.genome.gov/sequencing costs/). As a result, ~ 1.0 Gb genome can be sequenced at very low cost (e.g., approximately 1000 US dollars using Illumina Hiseq2000 series with 20–30× sequencing depth). Currently as of June 2018, the NCBI sequence read archive (SRA) database stores a total of 18,168 terabase (Tb) of NGS-derived DNA data (https://www.ncbi.nlm.nih.gov/sra/docs/sragrowth/). Such data size reflects an astonishing rate of NGS data production, which is 9.1 × 10^5^ times increase compared to the data amount as of June 2007. In contrast to the Sanger method, NGS technologies employ a wide array of chemical and/or biochemical disciplines for high throughput sequence production; pyrosequencing of Roche GS-FLX platform (Margulies et al. 2005), sequencing-by-synthesis of Illumina/Solexa Genome Analayzer (Bennett et al. 2005), sequencing-by-ligation of SOLiD Applied Biosystems (Milos 2008) and Polonator of Dover SystemsP, non-optical Ion Torrent sequencing using ion semiconductor (Life Science Inc.), single molecule real time (SMRT) sequencing or PacBio sequencing of the Pacific Bioscience (Eid et al. 2009) and Heliscope sequencer of Helicos Bioscience (Milos 2008). Of these, Illumina series of sequencing platforms has currently become the most predominant one (88%) in the genome sequencing market, followed by the GS-FLX platform (9%) by Roche (Kang et al. 2016a). In the past, bacterial artificial chromosome (BAC) library played a pivotal role for the whole genome sequencing (WGS), and BAC-by-BAC approach was frequently employed for the whole genome assembly. This method required a laborious process of physical map construction composed of numerous BAC clones. Before the PacBio technology was developed, BAC library construction was still a necessary step for the whole genome assembly. In the meantime, accuracy and length for reliable sequences by PacBio platform have been continuously improved, and the platform can actually generate long reads of 10–15 Kb with N50 value (Kang et al. 2016a). These long reads possess a superior advantage that can resolve a frequently encountered assembly problem of highly repetitive genomic regions. Thanks to such merits, solely NGS-based WGS is gradually becoming feasible through a combination of strategies, for example by producing a mixed length of sequence pools derived from short (Illumina)/medium (GS-FLX)/long read (PacBio)-generating platforms.

The emergence of various NGS sequencing platforms, along with the development of bioinformatics analysis tools, has highly accelerated production of fully or partially assembled whole genome sequences of many crop species. Currently (as of June 2018), a total of 140 genome accessions for land plants, which are derived from 95 species, are available at the NCBI genome database (https://www.ncbi.nlm.nih.gov/genome/browse#!/eukaryotes/). Out of 140 genome accessions, rice (*Oryza sativa*) genome accounts for the highest (14 accessions), followed by corn (*Zea mays*, 7 accessions). These reference or draft genome data have played a central role in producing and enriching other types of omics information; direct whole genome resequencing (WGR) for main purposes of discovering nucleotide variations followed by genome-wide association study (GWAS) and fabrication of SNP arrays, RNA sequencing for transcriptome analysis, genotyping by sequencing (GBS), methylome profiling for epigenomic analyses, small/long non-coding RNA profiling and Chip-seq analysis for DNA–protein interactions (Mochida and Shinozaki 2011).

### NGS-driven enrichment of genomic data

As mentioned, WGS information of major model or crop genomes can play a central role in translating genomic information across different species and expand its utility by producing other related omics data. Among those data, large scale genome resequencing would be one of the most predominantly conducted NGS-based research activities. Whole genome resequencing (WGR) is essential to reveal genome-wide nucleotide variations (representatively SNPs and InDels), which serve as the main resource for GWAS analyses. Many major crops, whose genomes are fully sequenced, have been re-sequenced, with different level of sequencing depth and coverage, mainly for the purposes of discovering genes and/or loci that are associated with traits of interest (Table 1). It is notable that most of the WGR data were produced extensively during the current decade, almost surely due to recent technical innovation and lowered cost of NGS data production. In the case of large scale WGR, hundreds of core accessions were re-sequenced and usually generated millions of nucleotide variations, which subsequently provide basic resources for the GWAS statistical analyses and fabrication of the SNP array chips. These WGR-based GWAS analyses appear to mainly focus on dissecting crop-specific traits beneficial for the domestication, such as large fruit/seed size, limited seed shattering and crop architecture in branching and stature (Table 1). In other cases, a small number of selected accessions, even only two parental lines, were re-sequenced mainly for the purpose of discovering genome-wide SNPs/InDels and developing genetic markers on a genome-wide scale (e.g., Jiang et al. 2017; Kevei et al. 2015; Kang et al. 2016b).

**Table 1:** List of selected WGR followed by GWAS/array-based identification of trait-associated loci in important crops

Species	Number of accessions	Sequencing depth	Nucleotide variations	Related traits and discovered loci	References
Soybean (*Glycine max*)	302	> 11×	9790744 SNPs, 876799 InDels	230 selective sweeps, 162 CNVs	Zhou et al. (2015)
	56	NA	5102244 SNPs	Seed coat color	Song et al. (2016)
	55	NA	5102244 SNPs, 707969 InDels	Domestication traits	Li et al. (2013)
	16	> 14×	~ 9 M SNPs	Domestication	Chung et al. (2014)
	246 RILs	~ 13.4×	463662–1004361 SNPs, 360544 InDels	Root-knot nematode resistance	Xu et al. (2013)
	28	14.8	541762 SNPs, 98922 InDels, 1093 CNVs	Genetic variation of Brazilian cultivars	dos Santos et al. (2016)
	14	30.3	242059 SNPs, 49276 InDels	Marker development	Song et al. (2015)
	165 MLs	NA (array)	104 selected SNPs	SMV resistance	Che et al. (2017)
Rice (*Oryza sativa*)	50	> 15×	6.5 M SNPs, 808 K InDels	Domestication	Xu et al. (2012)
	305	NA	NA	BADH 1 and 2, salt tolerance	He et al. (2015)
	391	NA	166418 SNPs	21 morphology traits, 11 grain quality, 10 root archetecture	Biscarini et al. (2016)
	132 RILs	4×	501499 SNPs	Yield-associated loci	Gao et al. (2013)
	270	NA	1019883 SNPs	Mesocotyl elongation	Wu et al. (2015)
	202	NA	NA	Chilling tolerance, 48 QTLs	Schläppi et al. (2017)
	3	43×	420475 SNPs, 95624 InDels	Yield-related genes	Jiang et al. (2017)
Maize (*Zea mays*)	278	~ 2×	27818705 SNPs	Domestication	Jiao et al. (2012)
	75	> 5×	21141953 SNPs	Domestication	Hufford et al. (2012)
Tomato (*Solanum lycopersicum*)	8	11.2×	> 4 M SNPs, 128000 InDels, 1686 CNVs	Breeding traits	Causse et al. (2013)
	60 RILs	~ 38×	4463846 SNPs	Meiotic recombination patterns	de Haas et al. (2017)
	2	40–44×	742963–6936608 SNPs, 149414–813246 InDels	Protein functions	Kevei et al. (2015)
Pepper (*Capsicum annuum*)	2	10×	6779745–7002670 SNPs	Bacterial wilt resistance	Kang et al. (2016b)
Cucumber (*Cucumis sativus*)	115	NA	3305010 SNPS, 336081 InDels	112 domestication sweeps	Qi et al. (2013)
Sesame (*Sesamum indicum*)	29	> 13	127347 SNPs, 17961 InDels	Control of flower number	Wang et al. (2014)
Watermelon (*Citrullus lanatus*)	20	4–16×	6784869 SNPs, 965006 InDels	Domestication	Guo et al. (2013)
Cotton (*Gossypium arboreum and G. herbaceum*)	243	~ 6×	17883108 SNPs, 2470515 InDels	98 associated loci for 11 agronomically important traits	Du et al. (2018)
Peach (*Prunus persica* L.)	129	~ 4.2×	4063377 SNPs	12 agronomic traits (e.g., fruit shape, non-acidity etc)	Cao et al. (2016)
Citrus (*Citrus* spp.)	111 varieties	NA (array)	1841 selected SNPs	17 quality traits of fruit (weight, shape, aroma intensity etc)	Minamikawa et al. (2017)

*NA* not available, *WGR* whole genome resequencing, *RIL* recombinant inbred line, *CNV* copy number variation, MLs mutant lines, *BADH* betaine aldehyde dehydrogenase, *SMV* soybean mosaic virus

Since the advent of NGS technology, to efficiently handle the ever-increasing massive amount of NGS-derived genomic data, the National Center for Biotechnology Information (NCBI) had launched NGS data-oriented DB, called Sequence Read Archive (SRA; https://www.ncbi.nlm.nih.gov/sra), in 2007, and has been offering interfaces for data submission, downloading and other genomic data-related services. On opposite side of the world, European Nucleotide Archive (ENA; https://www.ebi.ac.uk/ena/) has been providing similar services to the public. Typical genomic data statistic for major model and crops, which is currently available at SRA as of June 2018, is presented in Table 2. As shown, Arabidopsis and rice rank on the top in the number of SRA experiments, as the representative model or crop from the dicot and monocot plants, respectively. It is noteworthy that the SRA experiments have rapidly increased, compared to previous report by Mochida and Shinozaki (2011), ranging from several tens (e.g., soybean and potato) even to 1000 times (e.g., sorghum) depending on different species (Table 2). During the same time period (i.e., May 2011–June 2018), absolute amount of NGS data has increased approximately 140 times on average (https://www.ncbi.nlm.nih.gov/sra/docs/sragrowth/), which is comparable to above mentioned statistic. In other types of genomic data (e.g., transcriptome data, EST and 3-D structure of proteins), both species of Arabidopsis and rice also mark top rankers, which reflects their central roles as a TG language across monocot and dicot plant genomes.

**Table 2:** Omic-related statistics in major model and crop plants at NCBI (as of June 2018)

	Species name	SRA experiment	SRA fold increase^a^	GEO datasets	EST	Structure
Dicot	Thale cress (*A. thaliana*)	41,942	72.6×	53,885	–	1141
	Rape (*Brassica napus*)	4337	72.3×	762	6,43,881	9
	Field mustard (*Brassica rapa*)	2398	239.8×	814	2,14,482	3
	Soybean (*Glycine max*)	5705	35.7×	7457	–	148
	Common bean (*Phaseolus vularis*)	1326	15.9×	423	1,28,868	31
	Barrel medic (*Medicago truncatula*)	2221	18.8×	1791	2,69,501	44
	Tomato (*Solanum lycopersicum*)	6159	140×	2136	3,00,665	48
	Potato (*Solanum tuberosum*)	2657	55.4×	1702	2,50,140	53
	Grape (*Vitis vinifera*)	2959	134.5×	3762	4,46,678	26
Monocot	Rice (*Oryza sativa*)	51,332	67.6×	15,917	–	180
	Wheat (*Triticum aestivum*)	10,493	308.6×	3647	–	68
	Corn (*Zea mays*)	20,420	126.8×	11,306	–	178
	Barley (*Hordeum vulgare*)	2049	53.9×	2446	8,28,843	107
	Sorghum (*Sorghum bicolor*)	5127	1025.4×	673	2,09,835	13

*SRA* sequence read archive, *GEO* gene expression omnibus, *EST* expressed sequence tag

^a^Fold increases were compared with previous report by Mochida and Shinozaki (2011)

In addition to rapid innovative evolution of NGS technology itself, development and improvement of fully or semi-automated analytical tools/machines also significantly contribute to speeding up the rate of genome data accumulation. For example, the SNP Type assay platform, developed by Fluidigm (https://www.fluidigm.com/), has automated the PCR and following detection steps by employing integrated fluidic circuit (IFC) technology (Volpatti and Yetisen 2014), by which automatically mixes PCR reagents through the microfluidic channel networks. This automated platform can process 2304 (48 samples × 48 primers) to 9216 (96 × 96) PCR reactions and genotyping at a single run depending on IFC plates of choice. The Fragment Analyzer™ (Advanced Analytical Technologies Inc.; https://www.aati-us.com/instruments/fragment-analyzer/) allows high throughput genotyping of SSR markers using automated capillary electrophoresis system, and can process a maximum of 288 samples simultaneously. Furthermore, large scale resequencing data and resulting SNP/InDel information can provide a crucial basis for the application of array chip-based genotyping and GWAS analysis. Once sufficient amount data for the nucleotide variation are obtained, millions of SNPs and InDels can be fabricated into DNA chips, for representative examples Illumina Infinium HD (https://sapac.illumina.com/science/technology/) and Affymetrix Axiom (http://www.affymetrix.com/support/technical/). These array-based analytical platforms can be applied to the genome-wide analyses, such as GWAS, genotyping-by-sequencing (GBS) and QTL mapping.

### Genome comparative analysis as a central means for translational genomics

Comparative genomics (CG) provides a fundamental and practical means for translational genomics by making a flow of diverse genomic information from well-studied model system to relatively less-explored crop or orphan species. This allows us to characterize gene contents, to compare genomic architectures among individual species, and to explain structural similarities and/or differences between compared species within an evolutionary context, thereby enabling researchers to assess functional significance in genetic blueprints of each organism (Dong et al. 2004). The translatable information may include a wide range of data, such as cross-genome orthologous genes, genomic synteny and collinearity, evolutionary relationship among compared genomes, and epigenomic signatures. In general, degree of translational accuracy is proportional to closeness in evolutionary distances between multiple species in comparison. In the past, even still in the present, structural genomic comparison has been performed by employing genetic mapping with core genetic markers. In that case, researchers mainly focused on developing genetic markers and constructing genetic maps that were suitable for comparative analysis. For this purpose, gene-derived markers, which were relatively more conserved across different species, were successfully employed for macro-level genome conservation/divergence analyses (Choi et al. 2004; Ellwood et al. 2008; Phan et al. 2006). However, map-based comparative analysis has an obvious limit in its detail. Small number of markers, compared to the total number of genes that can be used for the comparison, may not reflect a full span of genomes under comparison.

Beyond the map-based comparative genome analyses, more systematic tools for genome-wide comparative analysis have been developed. For example, Artemis Comparison Tool (ACT; http://www.sanger.ac.uk/Software/ACT/) is a Java-applied software for visualizing comparative analysis (Carver et al. 2005, 2008) based on genome annotation by previously developed Artemis program (http://www.sanger.ac.uk/Software/Artemis/) (Berriman and Rutherford 2003; Rutherford et al. 2000). Artemis/ACT provides users with linear view of structural comparisons between two or more genomes and enables to explore synteny/collinearity and divergence among compared genomes. Different from Artemis/ACT, another program ‘Circos’, as implied in its name, displays the results of comparative analyses in a co-centric circular ideogram (Krzywinski et al. 2009). Not limited only to synteny-derived structural comparison of genomes, Circos can represent other various types of genome-wide data, such as nucleotide variation, GC contents, gene frequencies and more, and is capable of displaying such data using scatter plots, histograms, heat maps and lines. Circos is able to achieve large scale comparative analysis of multiple genomes by adopting the circular layouts and minimizing the inherent difficulties in visualizing complex genomic data (Krzywinski et al. 2009), and has become one of the most popular software packages for the genome comparative analyses. In particular, Circos has its strengths in effective/scalable illustration of genome positional relationships and flexible rearrangement of genomic components in the image.

**Fig. 1:**
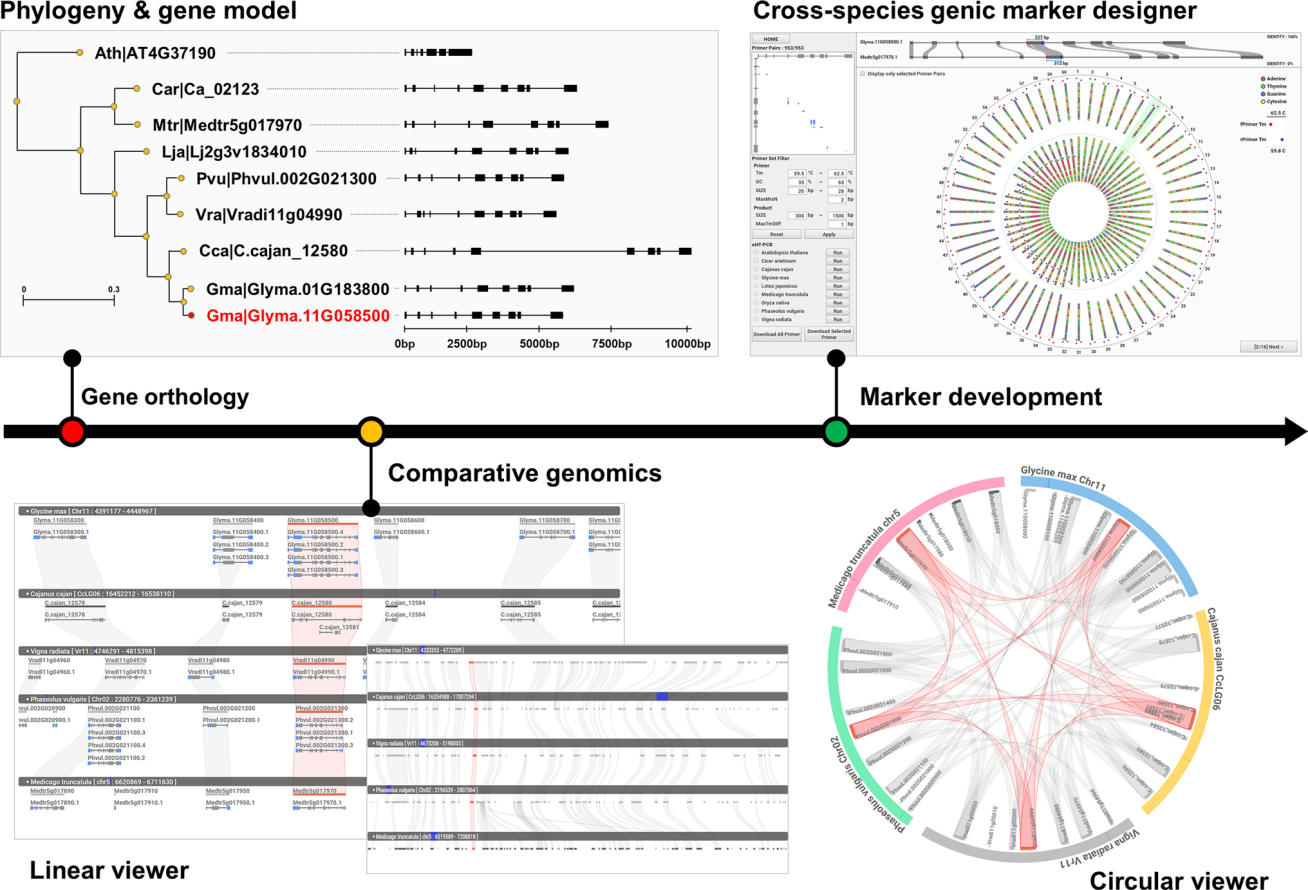
An example of customized bioinformatics platform for legume crop breeding. This figure represents a workflow from cross-species identification of orthologous genes to automated system for genic marker design, via genomic comparison on target genomic region (Glyma.11G058500 in this case) to reconfirm the orthologous relationship among genes. Linear and circular viewers depict structural and comparative analysis of orthologous genomic loci in five legume species. Lines and gray/colored boxes denote orthologous genes in different species

However, since above mentioned comparative analysis tools are all programs, suitable forms of data and resources should be pre-manipulated for the input and pipelined for further data processing to obtain the final results in visualized formats, which means that none of these two programs are the real time interactive platform for the comparative genome analysis. On the other side, database-linked bioinformatic systems are being developed to establish a real time-responsive comparative analysis platform. Figure 1 demonstrates one of those examples, which is an interactive platform dedicated to legume species (Choi et al. unpublished data). This platform pursues an integrative bioinformatics system in which DB and analysis tools/programs are all interconnected and interact together towards supporting genomics-based breeding design for legume crops. Although not shown for every component and module, comparative analysis interface, which is connected with corresponding genomic information, responds immediately in real time manner based on pre-calculated gene-to-gene orthology, and provides dual visualization options, i.e., linear and circular layouts (Fig. 1). In that way, one can exploit the system (e.g., identifying trait-associated orthologs in different species and finding syntenic regions of compared genomes) by using proper analysis options of user’s own choice.

### Bioinformatic resources for translational genomics and genetics

Databases (DB) and bioinformatic tools are essential for TG. There are currently a variety of DBs available worldwide with their own scientific missions. Since GenBank or NCBI (https://www.ncbi.nlm.nih.gov) had been officially established on the basis of the US Senate Legislative Agreement on support for NCBI in 1988, many other international or institutional DBs have been developed approximately for 30 year. Most of these DBs are publically and freely available to researchers and scientists, and provide a-click-away fast and easy access to genomic and other related biological information of researcher’s interest. In initial phase of DB development, each of DBs concentrated faithfully on their original mission with some limitation in target species and/or plant groups. Nevertheless, it recently appears that some of DBs intend to expand their missionary and functional scope, as more and more genomic data accumulate in the public sectors.

Typical plant-oriented DBs are represented in Table 3. Obviously, NCBI occupies the top rank in plant genome numbers stored in corresponding DBs with 288 land plants, followed by phytozome (93 plant genomes; https://phytozome.jgi.doe.gov/pz/portal.html) and PLAZA DBs (84 plant genomes; https://bioinformatics.psb.ugent.be/plaza/). These bioinformatic resources share some common features of DB equipped with genome browser, gene/sequence search and functional annotation, and genetic maps. Some of them are integrated with multiple species, while others are dedicated to a single one with species-specific mission for data mining (e.g., RiceXPro for rice transcriptome analysis). Among other single species-dedicated DBs, The Arabidopsis Information Resources (TAIR; https://www.arabidopsis.org/) should be the best and provides the most comprehensive A-to-Z contents of genomic information from gene functional annotation to transcriptome data as well as G-browser. For example, fully curated and functionally described genes of *A. thaliana* accounts for 13,822 [36.5% of 37,898 total number of genes (https://www.ncbi.nlm.nih.gov/genome/4)], compared to the second top 3334 genes of rice (Kang et al. 2016a). Although not dedicated only to plant genomes, UniProtKB (https://www.uniprot.org/) at ExPASy DB (https://www.expasy.org/) offers the most comprehensive and manually reviewed information on individual genes, which covers almost full scope of genomic/transcriptomic/proteomic data including 3D-protein structures. Currently as of June 2018, UniProtKB stores 557,491 manually annotated and reviewed entries (https://www.uniprot.org/statistics/), which should be the most refined data for pivotal roles in biological studies.

**Table 3:** Representative databases for plant genomes

	Resources	Database URL	Remarks and typical features	References
Multi-species DB	Phytozome	https://phytozome.jgi.doe.gov/pz/portal.html	93 plant genomes	Goodstein et al. (2012)
	Gramene	http://www.gramene.org/	44 plant genomes	Tello-Ruiz et al. (2018)
	PlantGDB	http://www.plantgdb.org/	27 plant genomes	Duvick et al. (2008)
	NCBI plant genome	https://www.ncbi.nlm.nih.gov/genome/	288 land plants	NA
	Ensembl plants	https://plants.ensembl.org/index.html	53 plant genomes	Aken et al. (2017)
	PLAZA	https://bioinformatics.psb.ugent.be/plaza/	55 dicots and 29 monocots	Proost et al. (2015)
	LIS (legume information system)	https://legumeinfo.org/	22 legume species	Dash et al. (2016)
	SGN (sol genomics network)	https://solgenomics.net/	6 Solanaceae species (tomato, potato, pepper, *N. benthamiana*, petunia, eggplant)	Fernandez-Pozo et al. (2015)
	GDR (genome databases for Rosaceae)	https://www.rosaceae.org/	21 Rosaceae species	Jung et al. (2014)
Single species-dedicated DB	TAIR	https://www.arabidopsis.org/	Arabidopsis (G-browser, gene ontology, synteny viewer)	Lamesch et al. (2012)
	Soybase	https://www.soybase.org/	Soybean (genetic map, G-browser, expression, mutant resources)	Grant et al. (2010)
	SoyKB	http://soykb.org/	Soybean (G-browser, traits, miRNA, metabolites)	Joshi et al. (2014)
	MtDB	http://www.medicagogenome.org/	*Medicago truncatula* (G-browser, annotation, genetic map)	Krishnakumar et al. (2014)
	CerealsDB	http://www.cerealsdb.uk.net/cerealgenomics/	Wheat (draft genome, array-based SNPs)	Wilkinson et al. (2016)
	MaizeGDB	https://www.maizegdb.org/	Maize (G-browser, SNPs, maps, genetic markers)	Andorf et al. (2016)
	RAP-DB	http://rapdb.dna.affrc.go.jp/	Rice (functional annotation, ortholog search)	Sakai et al. (2013)
	RiceXPro	http://ricexpro.dna.affrc.go.jp/	Rice (transcriptome-dedicated)	Sato et al. (2013)
	Oryzabase	https://shigen.nig.ac.jp/rice/oryzabase/	Rice (G-browser, genetic map)	Kurata and Yamazaki (2006)
	TFGD (tomato functional genomics database)	http://ted.bti.cornell.edu/	Tomato (transcriptome, metabolites, small RNA)	Fei et al. (2011)

*NA* not applicable

These DBs serves as useful platforms and/or interfaces, but within a limited scope and with specialized features, for the translational genomics study in plants. According to the time line, Phytozome, PLAZA and PlantGDB had emerged relatively recently, compared to longer-standing DBs such as TAIR, Gramene, SGN (Solanaceae Genomics Network; https://solgenomics.net/) and LIS (Legume Information System; https://legumeinfo.org/). All these DBs, except for TAIR, focus on comparative genome analyses across a wide range of green plants and are equipped with gene and genome-centric databases for cross-species translation. The goal of these DBs is to provide a platform transferring structural and functional information from model system to crops of agricultural and industrial importance. A growing number of reference genomes and NGS data are facilitating the enrichment of data contents, types and features along with the development/improvement of bioinformatics tools and algorithms. Some of DBs (e.g., Phytozome, Gramene, LIS etc) provide the application programming interface (API) that enable bioinformaticians to combine different sets of genomic data right on website without having to download the data, which can be implemented using various programing languages. In other cases, in-house-DBs are also used for the purposes of direct deposit and update of genome sequences.

Phytozome is a portal for the plant comparative genomics developed by the Department of Energy’s (DOE) Joint Genome Institute (JGI). Currently, the DB hosts 93 assembled and annotated genomes selected from 82 plant species (https://phytozome.jgi.doe.gov/pz/portal.html), and is on its way of integrating all those collections of data to facilitate accurate and comprehensive cross-genome translation. Towards this end, Phytozome has employed a combination of approaches (e.g., KEGG, ENZYME, Pathway, InterPro) and calculated inparanoid correlations of orthologs and paralogs for all annotated proteins in the database. Data search-by-query is offered by PhytoMine and BioMart, by which provide a template (i.e., certain type of defined genomic features) for data retrieval.

Among other genomic DBs, Gramene (http://www.gramene.org/) has published the latest report for its update (Tello-Ruiz et al. 2018). At its initial stage, Gramene began to develop the DB interface mainly with species from the grass family (i.e., rice as a nodal genome), but now has extended its scope into the dicot plants, thereafter including additional 23 eudicot species (http://ensembl.gramene.org/genome_browser/index.html). Currently, Gramene hosts a total of 44 reference genomes and > 2.0 million genes (more precisely 2,076,020 genes based on current DB statistics as of June 2018), most of which are organized into 62,367 gene families. Gramene operates in association with Ensembl Plants (https://plants.ensembl.org/index.html) and provides their shared features in appreciable parts, including data model, analysis workflow, whole genome alignments-based comparative analyses, synteny, phylogenetic trees, and other analytical tools such as BLAST, BioMart and the Variant Effect Predictor (VEP). Especially, Gramene offers an interactome analytical interface, called The Plant Reactome, for gene orthology-based projection to other genomes, in which rice genome play a central role for manual curation of interaction networks. Via the integration of all these data and a variety of analytical tools, Gramene has grown into one of the most integrative bioinformatic platforms providing in-depth genomic context, reactome pathway browser, expression profiles, comparative analyses and other useful analysis modules.

Legume Information System (LIS; https://legumeinfo.org/) is a legume-specialized web portal targeting for genomics/genetics-assisted breeding of legume crops. Among nearly 20,000 species, the third in flowering plants, genomic and genetic information of almost all 22 domesticated legume crops are hosted in LIS database (Dash et al. 2016). Of these 22 species, whole genome information for nine legumes is currently available, including *Medicago truncatula* (a central model) and *Glycine max* (the most important crop legume). Development of LIS started in 2001, and since then has been progressed through a collaborative effort between the National Center for Genome Resources (NCGR) and the USDA Agricultural Research Service (ARS) (Dash et al. 2016). Now, LIS has become a part of the federated management system (The Federated Plant Database Initiative for the Legumes; http://legumefederation.org) along with other legume-associated DBs (e.g., SoyBase, MtDB, Alfalfa Genome, PeanutBase). For purposes of wider spreading and broader sharing of genomic information with public sectors, LIS added Generic Model Organism Database (GMOD) as well as CMap and other general genome browsers (G- or J-browsers). Sharing the philosophy of the Legume Federation, LIS aims to accomplish a pan-legume translational platform by organizing and providing reference genome resources, information on genomic/genetic researches in legumes and visualized interface for the genome comparisons, with an intension of promoting legume crop breeding programs.

It seems that some DBs, more specifically single species-dedicated DBs but not limited to, tend to focus relatively more on trait-associated genetic/genomic data analyses, such as genetic markers/maps, QTL information, gene expression, DNA methylome and small RNAs (Table 3). Once WGS information is available and its relative cultivars or landraces are re-sequenced, genome-wide and high throughput development of molecular markers (e.g., SNP and SSR markers) is possible in a straightforward manner. Such NGS-derived molecular marker information is available and accessible in many DBs (e.g., Gramene, SoyBase, CerealDB). Recently, an enormous amount of > 20 million SNPs were produced from the rice 3000 genomes project (The 3000 Rice Genome Project 2014) and integrated into a new database, called SNP-Seek DB (http://snp-seek.irri.org/; Alexandrov et al. 2015). Because these SNP and InDel information is generated at random positions, it is not easy for users to pin-point genomic locations of nucleotide variations that may or may not be associated with traits of interest. PhytoMine (https://phytozome.jgi.doe.gov/phytomine/) presents a good example to solve such problem. By choosing a suitable combination of templates (e.g., ‘show the DNA sequence flanking specified gene’) in the query-based search tool of PhytoMine, one can readily make an access to the corresponding variations of interest.

Sometimes or frequently in fact, genomic data and bioinformatic tools are not readily accessible and analyzable for general users like breeding scientists mainly due to their complex nature and context, for which scientist need to learn program languages and software manipulating skills. To tackle these problems, many open resources for software libraries (e.g., Bioconductor and Bioperl) and web-based interfaces (EMBOSS and Galaxy) had been developed (Gentleman et al. 2004; Stajich et al. 2002; Rice et al. 2000; Goecks et al. 2010). Of these, Galaxy is the most recently developed open web-based platform and provides users with an interactive genomic workbench. Galaxy can operate, without any bioinformatics expertise, by making a data processing pipeline with selected analysis tools/softwares and chosen datasets (Goecks et al. 2010).

As mentioned thus far, it seems obvious that many of these DBs pursue the pan-genome translation across models and crops as an ultimate goal. Nevertheless, any of them are not completed towards this final mission. On the other side, a quite different approach has recently arisen. iPlant Collaborative (http://www.iplantcollaborative.org/), which was created and supported by the National Science Foundation (NSF) USA in 2008, is a representative example of probably the largest and community-driven open source-developing projects and provides an integrative and powerful cyberinfrastructure (CI or computational infrastructure) with the original purpose for plant and crop breeding (Goff et al. 2011; Merchant et al. 2016). Since then, iPlant CI has evolved into Cyverse to serve further expanded mission across all life science disciplines with an ambitious vision of ‘transforming science through data-driven discovery’ based on supercomputing capabilities (http://www.cyverse.org/). Towards this mission, Cyverse CI provides a synthetic and multi-layered structure in which consists of analysis tools, knowledge bases, data storage and management, workbench for computation and software adoption/development, collaborative network among communities, and more. For example, one of the functional layers, ‘the community-facing products’, can provide easy-to-use web access to interoperable applications, such as ‘Atmosphere’ (cloud computing CI), ‘Discovery Environment’ (web-based workbench for data analysis and management), ‘DNA Subway’ (configured workflow for genome analysis), and ‘Bisque’ (management, analysis and visualization of high throughput image data) (Merchant et al. 2016). Cyverse serves as a kind of marketplace where scientists share and distribute ideas on better tools, software, technologies and algorithms in the field of biological researches (Goff et al. 2011). Further development of Cyverse is community-dependent, and pursue self-evolving and sustainable open source platform by facilitating interdisciplinary collaborations among experts.

### Rationale and potential application of translational genomics to breeding design

**Fig. 2:**
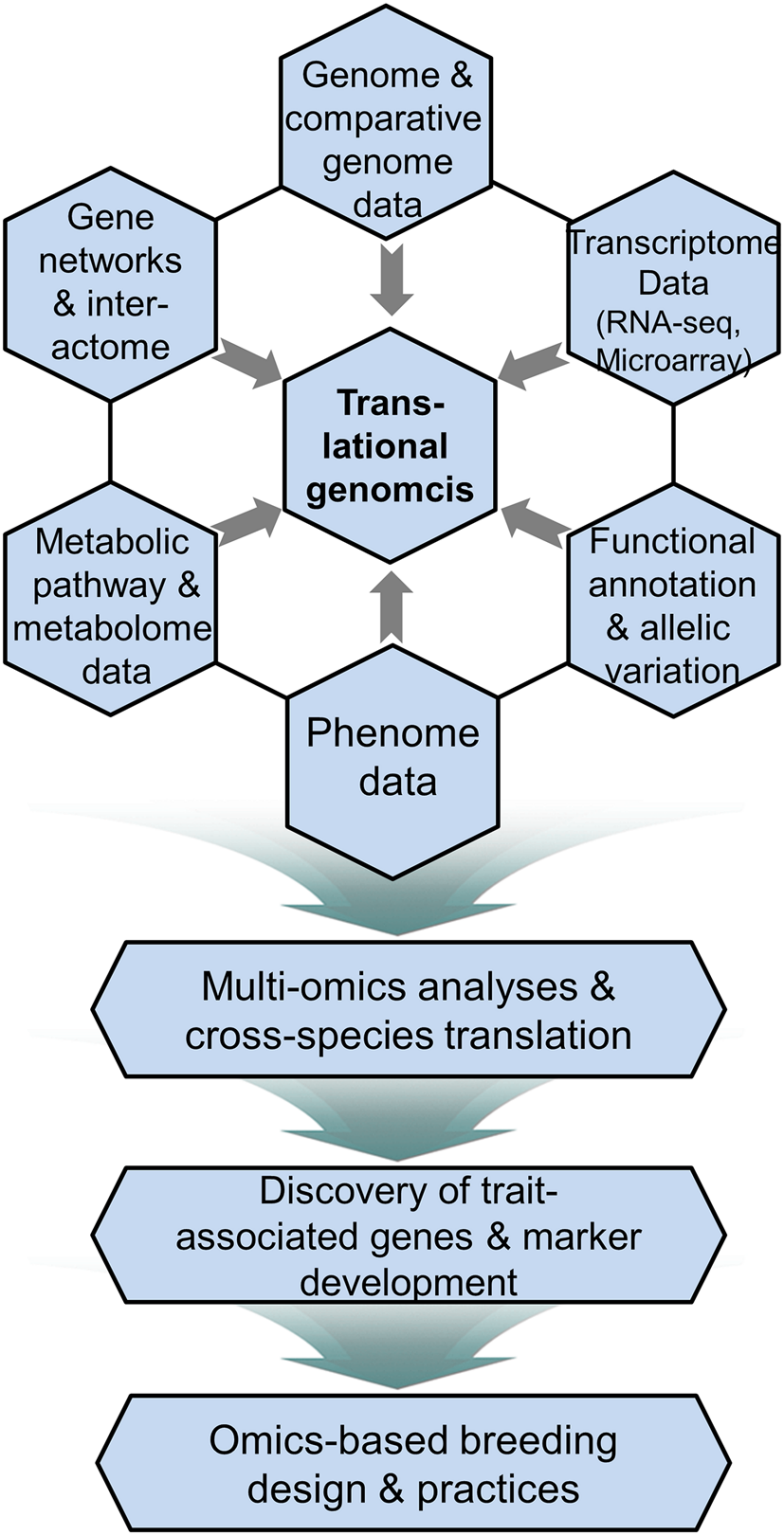
Schematic representation of multi-omics-based strategy for the crop breeding. The figure depicts that translational genomics plays a central role in omics-based breeding approaches and all these omics-integrated efforts converge into the discovery of trait-associated genes, alleles and marker development, which are the ultimate tools for the precision molecular breeding

TG-centered and multi-omics-integrated strategy for breeding processes is demonstrated in Fig. 2. Translation of genomic information is feasible based on the assumption that blue prints of all living organisms are written with the same chemical language system and genomic knowledge acquired from well-studied models can be projected onto other relatively less-studied crop genomes or orphan species. Such transfer of genomic information may occur at various levels, i.e., gene-to-gene, gene networks, whole genome-to-genome. Obviously, the translational accuracy is affected by evolutionary distances between species; the closer the distance between translated genomes, the more accurate the translated contexts of genomic information. In particular, orthology among translated genes is a strict prerequisite for translating the genomic contexts of specific interests in breeding of desired traits without any erroneous understanding. In order to properly accomplish this, orthologous relationships should be reconfirmed from multi-angled, at least three, analyses of genomic data. Firstly, homology-based identification of orthologs should be preceded as a basic and essential step. In addition to sequence homology, orthology of genes can be reconfirmed by their similarities in domain architectures, which are identified by the Hidden Markov Model (HMM) algorithm and can be searched at some specialized DBs, such as InterProScan (https://www.ebi.ac.uk/interpro/search/sequence-search; Hunter et al. 2012) and Pfam (http://pfam.xfam.org/search; Finn et al. 2014). Secondly, the orthologous relationships can be confirmed by phylogenetic analysis, because the homology is not always one-to-one relationship and, in reality, orthology is frequently confounded by paralogous genes that are generated by duplication during the evolutionary processes (Freeling 2009). Finally, orthology can further be reconfirmed within the context of syntenic relationships or gene collinearities in corresponding genomic regions in comparison (Paterson et al. 2010). Although chromosomes usually undergo the reshuffling and rearrangement of genomes after speciation, one can detect conserved genomic regions between species diverged from common ancestors and even among distantly related species, as well. Based on those synteny analyses, QTL information (involving multiple genes for the QTL traits) of nodal crops, beyond simple gene-to-gene translation, could be transferred to other orphan, but phylogenetically related, crops.

Transcriptome data can also play a pivotal role in identifying orthologous genes in other species by carefully investigating expressional behavior under regulated conditions of intended experimental settings. Co-expressed genes with the same or similar functional annotations across different genomes may form a gene network and give key information on predictable dynamic interactions among genes and proteins (Lee et al. 2010). This approach may allow us to obtain a broader and higher level of insight into the orchestrated biological mechanisms occurring in living organisms that are actively respond to changing environments and given stresses. A combination of co-expression pattern of genes and data for protein-to-protein interactome (PPI) may create a synergy for more accurate and higher predictability within the interacting networks. AraNet (http://www.functionalnet.org/aranet/) is such a good example for functional gene network analysis dedicated to *A. thaliana*, but transferrable to other species, in which different types of omics data are integrated by modified Bayesian algorithm and interlinked based on probabilistic log-likelihood score (LLS) representing a functional linkage between interacting genes (Lee et al. 2010). If a reliable key network is extracted from the multi-omics-integrated DBs, cross-genome translation would be feasible at the gene network and/or interactome levels. In this case, one will need to take into consideration of genetic/genomic background of each organism for the expectation of translational efficiency, because functional criteria of individual genes or networks are orchestrated by the whole genomic context of given species.

Similarly, metabolic/biochemical pathways and resulting metabolic profiles could be inter-transferred between different species. Metabolomics is one of the important and extended omics layers right beyond the central dogma (i.e., covering from polynucleotide chains to resulting proteins), and can provide a direct chemical evidence by which allow us to dissect the phenomenal aspects occurring in the cells or tissues under certain natural conditions or experimentally regulated settings. It is generally known that key players for the metabolome formation (i.e., enzymes) occupies approximately a half of all encoded genes, which is the largest portion of all functional proteins, and thus can explain an appreciable portion of the entire biological processes. Moreover, massive production of the metabolome data has been increasingly accelerated due to the technical advancement of ultra-performance liquid chromatography and tandem quadrupole mass spectrometry (Sawada et al. 2009). In addition, many useful DBs are available for analyses of metabolic pathways, including KEGG (https://www.genome.jp/kegg/; Kanehisa et al. 2014) as a general platform and the ‘Plant Metabolic Network’ (PMN; https://www.plantcyc.org/) for plant-dedicated metabolic pathways. Especially, the PMN is a combined pathway-dedicated DB and currently hosts 77 plant species-specific metabolic pathways including Arabidopsis, rice, soybean, papaya and many more. Presence/absence and variation/modification of enzymes between compared metabolic pathways may determine their genetic and functional features corresponding to different species or genotypes. Furthermore, metabolome profiling is useful to evaluate metabolic phenotypes and to analyze metabolite quantitative trait loci (mQTL) in natural or segregation populations (Mochida and Shinozaki 2011). Taken together, comparative pathway analysis and metabolome profiling would be able to make a synergistic effect on breeding a trait associated with production of functionally useful metabolites in crops.

In recent years, GWAS analysis has been widely used to discover genes, genomic loci and SNP/InDel that are associated with useful crop traits of interest. Beyond the capability of genetic map-based QTL analyses in the past, re-sequencing and/or array-based GWAS is making it possible to a lot more precisely predict or identify the alleles directly linked to certain phenotypic features for breeding, thereby resulting in revelation of trait-associated single/a few or a combination of nucleotide variations. Many cases of GWAS/array-based identification of trait-associated genomic loci in crop plants are shown in Table 1. Additionally, development of high-throughput phenotyping system (HTPS) is important to facilitate systematic phenotype-linked genomic analyses. Yang et al. (2014) reported a successful case of HTPS-integrated GWAS approach in rice. As a result, they could identify a total of 141 genomic loci associated with 15 defined agronomic traits, of which 25 loci contained genes that were previously known for their functions (Yang et al. 2014). Subsequently, these phenotype-linked variations can be developed into trait-associated genetic markers, which are very useful molecular tracer for breeders, and these markers can serve as a powerful tool for the genomics-assisted precision breeding. Furthermore, the phenotype-associated genomic information could be translated into other related plant or crop genomes, wherever possible, based on the syntenic relationships. Via these multi-angled and omics-driven approaches, translation of cross-species phenotypic annotation associated with complex traits would be feasible, and become more precise as the omics data are more completely integrated.

## Conclusions and perspectives

Due to the advent of the NGS technologies and phenomenal growth of genomics and other omics data, now it seems doubtless that we are facing a big move into the era of ‘bio-big-data’. These technical innovations have driven and led to the production of genomic information of many reference genomes, resequencing of numerous crop accessions, RNA-sequencing for transcriptomes and many others. Integration of all those genome-wide information may create novel in-depth molecular signatures bridging the genomic variations found in the omics study with corresponding phenotypes of complex traits, which were not readily handled in the past. Such genomic-to-phenotypic correlations could be translated among plant genomes via homology-based or synteny-based information transfer. It is strongly expected that TG approach will improve and accelerate the modern breeding processes, compared to the conventional breeding programs that still remain the mainstay but are relatively time-consuming and labor-intensive. Towards this direction, integrative omics approaches will collectively serve towards the precision breeding through which enable breeders to elaborate target traits into a crop of desire.

However, without comprehensive information for a diverse array of well-defined phenotypic features (or phenotypic ontology; PO), omics-derived big data could not be properly applied for the precision breeding. Thus, in order for successful application of TG strategies in the future, following issues should be taken into consideration. First, cost-effective and precise phenomics facilities or platforms should be equipped to interconnect corresponding information between genomic and phenotypic data. Second, sustainable and integrative data management system (e.g., The Integrated Breeding Platform; www. integratedbreeding.net) need to be established to synthetically and efficiently manage all breeding-related data and field activities. Third, a lot more trait-associated markers tagged with phenotypic annotations should be developed to allow easy and direct applications for on-site breeding practices. Fourth, institutional collection and systematic organization of diverse germplasms for cultivars, wild types and mutant lines need to be accomplished to facilitate purpose-driven selection of plant resources for production of corresponding omics data. In addition, these resources need to be freely shared among breeders and genomics scientists. Fifth, computational/bioinformatic tools and more efficient algorithms need to be further developed to meet ever-increasing data size and analysis capabilities in up-coming future. It is also anticipated, at certain time point in the future, that introduction of artificial intelligence (AI)-combined platform, which is deep-learned with the ‘bio-big-data’, would be possible as an ultimate form of omics-based breeding program. Finally, by taking together all the genomic and phenomic information, platform for the genomic selection (GS) need to be prepared as a practical translational breeding pipeline. GS operates by genome-wide marker profiles and allows to predict breeding outcome by projecting the ‘genomic estimated breeding value (GEBV)’ of the training population to breeding candidate population, thereby enabling to select suitable breeding lines based on overall phenotypic performance of crops.

In addition to above mentioned omics approaches, other layers of omics disciplines, including epigenome, regulome (ome of regulation-involved DNA/RNA elements), hormonome and promotome (ome of promoter elements) may need to be further integrated to gain knowledge based on entire breadth of omics data. Such multi-omics-driven systems approach will allow us to facilitate overall breeding processes and lead us to the final stage of the breeding program, so called ‘designable/predictable breeding’ or ‘reverse breeding’.
